# NF45 and NF90 Bind HIV-1 RNA and Modulate HIV Gene Expression

**DOI:** 10.3390/v8020047

**Published:** 2016-02-16

**Authors:** Yan Li, Michael Belshan

**Affiliations:** 1 Department of Medical Microbiology and Immunology, Creighton University, Omaha, NE 68178, USA; yanli@creighton.edu; 2 The Nebraska Center for Virology, University of Nebraska, Lincoln, NE 68583, USA

**Keywords:** HIV, NF45, NF90, transcription, RNA regulation

## Abstract

A previous proteomic screen in our laboratory identified nuclear factor 45 (NF45) and nuclear factor 90 (NF90) as potential cellular factors involved in human immunodeficiency virus type 1 (HIV-1) replication. Both are RNA binding proteins that regulate gene expression; and NF90 has been shown to regulate the expression of cyclin T1 which is required for Tat-dependent *trans*-activation of viral gene expression. In this study the roles of NF45 and NF90 in HIV replication were investigated through overexpression studies. Ectopic expression of either factor potentiated HIV infection, gene expression, and virus production. Deletion of the RNA binding domains of NF45 and NF90 diminished the enhancement of HIV infection and gene expression. Both proteins were found to interact with the HIV RNA. RNA decay assays demonstrated that NF90, but not NF45, increased the half-life of the HIV RNA. Overall, these studies indicate that both NF45 and NF90 potentiate HIV infection through their RNA binding domains.

## 1. Introduction

Like all viruses, human immunodeficiency virus (HIV) requires host cell factors for productive replication. HIV produces only 15 proteins but replicates in several cell lineages and exhibits a complex life cycle [1]. In addition, the host cell elicits an innate response to defend against viral infection and spread. Characterization of both the pro- and anti-viral factors during infection may discover new targets for the development of novel therapeutic strategies that inhibit virus replication and/or pathogenesis. A number of genetic and proteomic studies have been undertaken to identify cellular factors involved in HIV replication and the current National Institutes of Health (NIH) interaction database contains greater than 3000 proteins [2]. Given the size of the database, it is disappointing that only one antiviral compound targeting a cell factor, Maroviroc, has been approved for clinical use as an anti-viral inhibitor [3].

**Figure 1:**
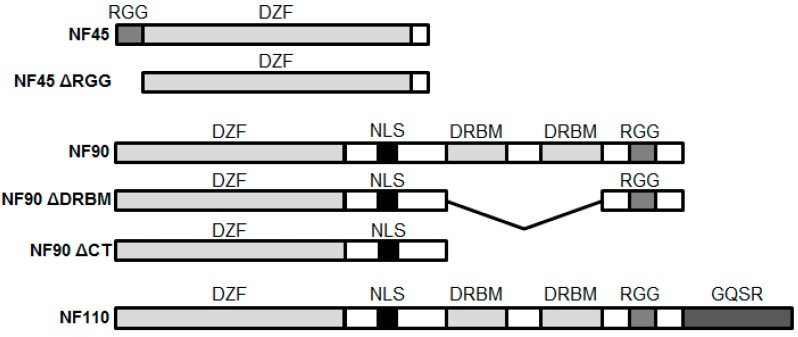
Schematic structure of NF45, NF90, NF110 and the mutants used in this study. Domain abbreviations include: RGG, arginine-glycine-glycine domain; DZF, domain associated with zinc fingers; NLS, nuclear localization signal; GQSR motif, glycine, glutamine, serine, arginine motif.

Our laboratory has completed a number of proteomic screens for HIV factors [4,5,6]. Nuclear factor 45 and nuclear factor 90 (NF45 and NF90, respectively) were identified as candidate HIV factors in a screen of HIV nucleoprotein complexes [5]. These proteins are the products of the interleukin enhancer binding factor 2 and 3 genes (ILF2 and 3, respectively). Both proteins participate extensively in RNA metabolism, including the regulation of messenger, noncoding, and regulatory RNAs. NF45 and NF90 are distinct proteins, but share common domains (Figure 1). NF45 has an N-terminal arginine- and glycine rich (RGG) domain at position 3–22, and a domain associated with zinc fingers (DZF) at position 104–338. NF90 has an N-terminal DZF and C-terminal RGG domain. It also has a defined nuclear localization signal as well as two double stranded RNA binding (DRBM) motifs. In addition to their individual functions, NF45 and NF90 can heterodimerize via their DZF domains to form a RNA regulatory complex. The NF45-NF90 complex has been shown to interact with DNA-dependent protein kinases (DNA-PK) and be involved in protein kinase C mediated DNA damage repair [7]. Both proteins also bind the interleukin (IL)-13 promoter HS-4 region and increase IL-13 expression during T cell activation [8]. Recently, NF45 and NF90 are found to be the components of precursors to 60S (pre-60S) ribosomal subunits [9]. NF45 has been shown to regulate the activity of NF90, and the knockdown of NF90 destabilizes NF45 expression [9,10]. Alternative splicing of NF90 produces a larger isoform, NF110, which contains an altered C-terminus with a distinct GQSR motif (Figure 1). The C-terminal domain of NF110 has been shown to enhance Survivin expression [11], but overall the functions of NF110 are less well understood. Similar to NF90 it binds RNA and can enhance gene expression, although it appears to be bound to chromatin in the cell [12,13].

Efficient HIV gene expression requires hyperphosphorylation of RNA polymerase II by the Positive Transcription Elongation Factor b (P-TEFb), a complex of CDK9 and cyclin T1 [14,15,16,17]. Recruitment of P-TEFb is accomplished by the HIV regulatory protein Tat, which binds the small step-loop *trans*-activation response element region (TAR) in the 5′ end of the new synthesized viral RNA. NF90 has been identified as a regulator of HIV transcription by modulating cyclin T1 expression. Cyclin T1 expression requires binding of NF90 to the 3′ UTR of its mRNA. Therefore, knockdown of NF90 decreases HIV gene expression by reducing cyclin T1 levels [18]. Overexpression of NF90 can increase HIV promoter activity when co-transfected with an HIV reporter virus [13,18]. Other studies have investigated the role of a C-terminal mutant of NF90 (NF90ctv). Expression of this mutant disrupts Tat-dependent transcription by competitive binding of the TAR element [19]. It also interacts with HIV Rev and inhibits its RNA export activity [20]. Although a later study demonstrated that NF90ctv facilitated the export of *gag* mRNA in a Rev-independent manner [21]. However, the NF90ctv isoform is not expressed in cells and may not be biologically relevant [18,22].

Combined, previous studies show that NF90 is required for HIV gene expression by regulating cyclin T1 expression. In this study, we further investigated the roles of NF45 and NF90 in HIV replication using overexpression studies. Our results show that both proteins promote HIV infection. The RNA binding domains were required for the effect as deletion of RGG domain in NF45 or deletion of DRBMs in NF90 reduced any potentiation. Notably, the overexpression of either protein did not affect Cyclin T1 levels in cells. NF45 and NF90 were also found to bind HIV RNA *in vitro*, although the interactions appear to be sequence independent. Finally, RNA stability assays demonstrated that NF90 increased the half-life of HIV RNA, suggesting a second mechanism for the enhancement of HIV gene expression. Combined with previous studies, these results show NF45 and NF90 can potentiate HIV infection and define additional mechanisms by which NF45 and NF90 modulate HIV gene expression.

## 2. Materials and Methods

### 2.1. Cell Culture

293T and HeLa Tet-On^®^ 3G cells (Clontech Laboratories, Mountain View, CA, USA) were grown in Dulbecco’s modified Eagle’s medium (DMEM) supplemented with 10% Fetal Clone III (Hyclone, Logan, UT, USA), 100 U/mL penicillin and 100 µg/mL streptomycin. The media for the HeLa Tet-On^®^ 3G also contained 100 μg/mL G418. All cells were grown at 37 °C with 5% CO_2_ in humidified incubator.

### 2.2. Proviral Clones and Expression Plasmids

The following plasmids have been described previously: pNLX [23], pNL4-3-Luc [24,25], pSV2tat72 [26] and pBlue3′LTR-Luc [27,28]. pCMV-NF45 was purchased from OriGene (OriGene, Rockville, MD, USA). pEF6-NF90 was constructed by subcloning the NF90 ORF from pCMV-Sport6 (Thermo Fisher Scientific Inc., Waltham, MA, USA) into pEF6-TOPO by TOPO cloning (Life Technologies, Carlsbad, CA, USA) using primers 5′-ATGCGTCCAATGCGAATTTTTGTGAATG-3′ and 5′-CTAGGAAGACCCAAAATCATGATAG-3′. pTarget-NF45 ΔRGG was constructed by amplifying a truncated version of NF45 with the RGG region deleted and an artificial start codon (underlined) inserted (primers: 5′-ACCATGTTCAGGCCCTTTG-3′ and 5′-CTAGGAAGACCCAAAATCATGATAG-3′). The amplicon was TA cloned into pTarget (Promega, Madison, WI, USA). pTarget-NF90 ΔCT was similarly subcloned from pEF6-NF90 using 5′-ATGCGTCCAATGCGAATTTTTGTGAATG-3′ and a 3′ primer with a premature stop codon (underlined; 5′-TTACCCGTCCTCCTCCATTGGGCGTTT-3′). The deletion mutant, pTarget-NF90 ΔDRBM, was constructed by PCR overlap mutagenesis using the overlapping primers 5′-GGGCGAGAGGGGTGTCTGCCTTCTCCTCTTTCT-3′ and 5′-AGAAAGAGGAGAAGGCAGACACCCCTCTCGCCC-3′.

pET28a(+)-NF45 and NF90 were generated by inserting NF45 and NF90 cDNA fragments into pET28a EcoRI and NotI sites. pTRE-LTR was made by subcloning HIV LTR and R3 region using primers 5′-CGGGATCCTTTTGCCTGTACTGGG-3′ and 5′-CGACGCGTTGCTAGAGATTTTCCACA-3′. pGEM-T-IL2 was cloned from 293T cell RNA using standard cloning techniques (primers: 5′-GCCTTCTATTTATTTAAATAT-3′ and 5′-TTATCAAATTTATTAAATAGTT-3′). All the plasmids used in this study were verified by sequencing using the Creighton University biomedical core facility sequencing service.

### 2.3. Viral Assays

Viruses were prepared and normalized for infectivity as described previously [4,29]. For HIV infection assays, 293T cells (~5 × 10^4^) were seeded in 24 well plates one day prior to transfection. For overexpression experiment, cells were transfected with total 0.4 µg expression constructs or control pcDNA3.1 0.4 µg vector using 1 mg/mL polyethylenimine (PEI). For knockdown experiment, cells were transfected with small interfering RNAs (siRNAs) targets to NF45 and NF90 (Santa Cruz Biotechnology, Santa Cruz, CA, USA) at 1 ng/well in 6-well plates using Genmute reagent as directed by the manufacturer (SignaGen, Gaithersburg, MD, USA). At 24 h post-transfection cells were infected with virus for 4 h and the media changed. At 48 hours post-infection (hpi) the cells were lysed with M-PER (Mammalian Protein Extraction Reagent; Thermo Fisher Scientific). The lysates were cleared by centrifugation at 10,000× *g* for 4 min at 4 °C and luciferase (Luc) activity measured using ONE-Glo™ Luciferase reagent and a GloMax^®^-96 Microplate Luminometer as directed by the manufacturer (Promega). The luminometer reading (RLU) for each sample was normalized to its total protein concentration. To measure virus release from transduced cells, 1 × 10^5^ 293T cells were seeded in 6-well plates one day prior to transfection with 0.5 µg pCMV-NF45 or pEF6-NF90. At 24 h post-transfection cells were infected with HIV pseudotyped with the vesicular stomatitis virus glycoprotein (VSVg) envelope for 4 h and the media changed. For the measurement of virus release from cells co-transfected with the factors, cells were transfected with 0.5 µg pCMV-NF45, pEF6-NF90 plus 0.1 µg pNLX overnight followed by a media change. Cell supernatants were collected at 8 h post media change in both experiments. Virus release was measured by *in vitro* reverse transcription assays as previously described [30]. For virus expression assays each culture was transfected with expression construct and control vector pCDNA3.1 0.4 µg plus 0.1 µg pNL4-3-Luc. At 48 h post-transfection, cells were lysed and analyzed for Luc activity. MTT assays were performed using the CellTiter 96 non-radioactive cell proliferation assay according to the manufacturer’s instructions (Promega).

### 2.4. Immunobloting

Cell samples were lysed and mixed 1:1 with 2× sodium dodecyl sulfate polyacrylamide gel electrophoresis (SDS-PAGE) loading buffer, boiled for 10 min and separated by SDS-PAGE. Proteins were transferred to polyvinylidene fluoride (PVDF) and detected by Western blot using the following primary antibodies: anti-NF45 (A-8), anti-actin (I-19) and anti-cyclin T1 (T-18) were all obtained from Santa Cruz Biotechnology; anti-FLAG (M2) was from Sigma (St. Louis, MO, USA) and the anti-NF90 antibody was from Abgent (San Diego, CA, USA). Primary antibody incubations were 1 h, except the detection of the NF90 deletion mutants with the Abgent antibody required an increased length of incubation (>3 h). Incubation (30 min) with HRP conjugated anti-rabbit, anti-mouse IgG secondary (GE Healthcare, Piscataway, NJ, USA) or anti-goat IgG secondary (Sigma) antibody was used to detect primary antibody binding, which was visualized by chemiluminescent staining (Pierce Biotechnology, Waltham, MA, USA). Images were captured using radiographic film, scanned to computer, adjusted for brightness and contrast if necessary, and cropped for size.

### 2.5. Immunoprecipitation-RT-PCR Assays

Immunoprecipitation-RT-PCR (IP-RT-PCR) assays were performed essentially as described previously [31]. The cells were transfected 24 h prior to infection. At 24 hpi cells were washed and lysed with RIPA buffer (50 mM Tris-HCl pH 7.4, 150 mM NaCl, 1% Triton x-100, 1% Sodium deoxycholate, 0.2% SDS and 1 mM EDTA). Cell lysates were precleared with agarose beads, then incubated with anti-FLAG M2 magnetic beads (Sigma-Aldrich, St. Louis, MO, USA) for 12 h at 4 °C with rotation. Beads were washed 4 times with RIPA buffer and split into two tubes—one for immunoblots and one for RNA isolation. The samples for RNA were treated with proteinase K for 30 min at 37 °C and RNA isolated by acid phenol-chloroform extraction. RNA was reverse transcribed using MLV RNA reverse transcriptase, and DNA quantified by real-time PCR with *gag*-specific primers (5′-GAAGCGCGCACGGCAAGAGGC-3′ and 5′-GCACACAATAGAGGACTGCTATTGTA-3′), using iTaq Universal SYBR Green Supermix and a Bio-Rad CFX Connect Real-Time PCR detection system.

### 2.6. Purification of Recombination NF45 and NF90 Proteins and RNA Cross-Linking Assays

Recombinant NF45 and NF90 proteins (rNF45 and rNF90) were expressed in BL21 *Escherichia coli* (*E. coli*) cells (Novagen, Madison, WI, USA). Harvested cells were lysed by EmulsiFlex-C3 (AVESTIN, Ottawa, ON, USA), and His-tagged fusion proteins were purified under native conditions using HisPur Cobalt Resin (Thermo Fisher Scientific) following the manufacturer’s protocol. The purity of fusion proteins was confirmed by SDS-PAGE followed by Coomassie staining. Purified rNF45 and rNF90 proteins were dialyzed and stored in 50 mM Tris-HCl (pH 8.0), 50 mM NaCl and 10% Glycerol at −80 °C. The DNA templates for RNA probe production contained the T7 primer sequence linked to the HIV target sequences. These were synthesized by PCR using a pNLX template or pGEMT-IL2 template. Primers were as follows: U3 part 1 (sense 5′-TAATACGACTCACTATAGGGTGGAAGGGCTAATTTGG-3′ and antisense 5′-ATGCTGGCTCATAGGGTGTAACAAG-3′) , U3 part 2 (sense 5′-TAATACGACTCACTATAGGGGCTTGTTACACCCTATG-3′ and antisense 5′-CCAGTACAGGCAAAAAGCAGCTGC-3′), R (sense 5′-TAATACGACTCACTATAGGGGGTCTCTGGTTAGA-3′ and antisense 5′-TGAGCACTCAAGGCAAGCTTTATTG-3′), U5 (sense 5′-TAATACGACTTCACTATAGGGAAGTAGTGTGTGCCCCG-3′ and antisense 5′-CTGCTAGAGTTTTCCACACTGACT-3′) and IL-2 3′mRNA (sense 5′-TAATACGACTCACTATAGGGGCCTTCTATTTATTAAATAT and antisense 5′-TTATCAAATTTATTAAATAGTT-3′).

RNA was generated by *in vitro* T7 transcription (New England Biolabs, Ipswich, MA, USA) in the presence of [α-^32^P]UTP. Transcribed radiolabeled RNA was purified with a RNA probe purification kit (Omega Bio-Tek, Norcross, GA, USA), denatured at 80 °C for 10 min in 20 mM Tris-HCl, pH 7.5, 100 mM NaCl, annealed by slow cooling, and stored at –80 °C. For RNA-binding reactions, 2 µg purified GST, rNF45 or rNF90 protein was incubated with probes in binding buffer (100 mM KCl, 1 mM MgCl2, 0.5 mM EDTA, 1 mM dithiothreitol, 50 g/mL yeast tRNA, and 10% glycerol) for 30 min at 37 °C. A probe alone group was used as a negative control. After incubation, reaction samples were irradiated with 4 × 10^5^ J using UV Stratalinker 1800 (Stratagene, La Jolla, CA, USA). Samples were treated with 0.1 mg/mL RNase A at 37 °C for 30 min, the reaction terminated by boiling 5 min in an equal volume of 2× sample buffer, and separated by 12% SDS-PAGE. Gels were dried, exposed to phosphorimager screens overnight and imaged using a Typhoon 9400 phosphorimager (GE Healthcare).

### 2.7. RNA Decay Assays

The transfection and selection of the pTRE-LTR HeLa Tet-On^®^ 3G Cell Line (HeLa-iLTR) was performed following the manufacturer’s instructions (Clontech). For RNA decay assays, 1 × 10^5^ HeLa-iLTR cells were seeded into 6-well plates 24 h prior to transfection with 1 µg pCDNA3.1, pCMV-NF45 or pEF6-NF90 using GenJet™ transfection reagent (SignaGen Laboratories, Rockville, MD, USA) following the manufacturer’s instructions. At 24 h post transfection 10 ng/mL of doxycycline was added to induce expression of HIV RNA. After 12 h of induction, the cells were washed 3 times and returned to cDMEM. Total RNA was harvested at 0, 8, 16, 24 and 32 h (Omega Biotech, Norcross, GA, USA). RNA was reverse transcribed using random primers and cDNA was amplified using primers (5′-TCTGGCTAACTAGGGAACCCAC-3″ and 5′-CTGACTAAAAGGGTCTGAGG-3′). Primers designed to 18S RNA were used as an internal control (5′-GTAACCCGTTGAACCCCATT-3′ and 5′-CCATCCAATCGGTAGTAGCG-3′).

## 3. Results

### 3.1. The Overexpression of NF45 or NF90 Increases HIV Infection and Virus Production

**Figure 2:**
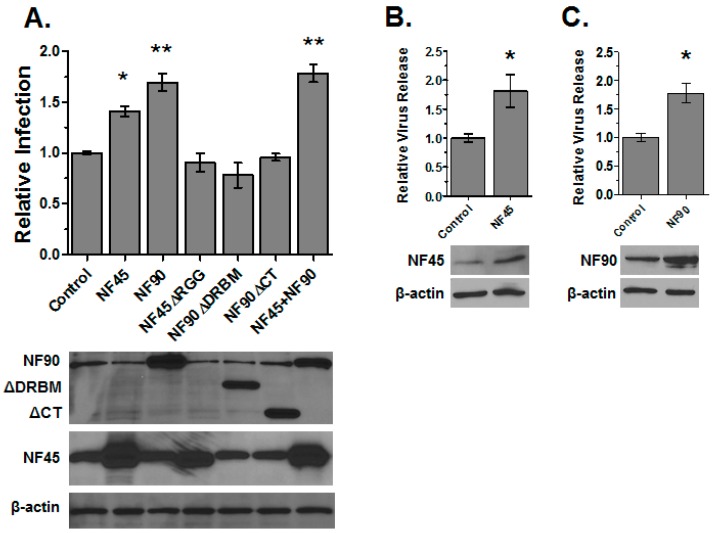
NF45 and NF90 potentiate human immunodeficiency virus (HIV) infection. (**A**) 293T cells were transfected with the indicated expression vectors or pcDNA3.1 empty vector as a control. After 24 h the cells were infected with vesicular stomatitis virus glycoprotein (VSVg)-pseudotyped HIV-Luc reporter virus. At 24 h post-infection (hpi), the cells were harvested and infection quantified by Luc assay and normalized to protein concentration. Results in this, and all other panels are presented relative to the control group. Error bars in this and all other figures represent standard error of the mean. Protein expression was monitored by immunoblot using β-actin as a control (bottom panels); (**B**,**C**) Single cycle production assays for NF45 and NF90, respectively. Cells pre-transfected with plasmids to indicated factors for 24 h were transduced with HIV pseudotyped with VSVg. After 4 h the media was changed and the cells incubated an additional 8 h. Cell supernatants were collected and virus production measured by *in vitro* reverse transcriptase assay as described in Materials and Methods. Plots in all panels show the total data from three independent experiments with triplicate transfections; blots are representative of all three experiments. (* *p* < 0.05 and ** *p* < 0.01 by two-tailed *t*-test in this and all figures).

In a previous proteomic study of HIV reverse transcription and preintegration complexes we observed changes in the expression of NF45 and NF90 in HIV infected cells by mass spectrometry [5]. Since both NF45 and NF90 are RNA binding proteins, we speculated that they might participate in the early steps of the HIV life cycle. Our initial approach was to examine the effect of over-expressing each protein on HIV infection. Cells were transfected with individual expression constructs 24 h prior to transduction with a HIV luciferase reporter virus (HIV-Luc) pseudotyped with the VSVg. Overexpression of NF45 led to a modest increase in Luc expression, indicating it enhanced infection (Figure 2A). NF45 has been shown to interact with the RNA of several viruses [32,33], leading us to hypothesize that it interacted with HIV RNA. To initially test this hypothesis, we tested if deleting the RNA binding domain of NF45 would mitigate its ability to enhance HIV infection. A mutant with a deletion in the N-terminal RGG domain was constructed (ΔRGG) and tested in infection experiments. Deletion of the RGG domain ablated the ability of NF45 to enhance HIV infection (Figure 2A). To confirm the enhancement effect of NF45 we repeated the infection experiments with wild-type non-pseudotyped HIV and measured the production of virus from infected cells by reverse transcription assay (Figure 2B). Similar to the Luc assays, a small, but significant increase in HIV infection occurred in cells over-expressing NF45.

Next we investigated NF90. Knockdown of NF90 by RNA interference reduces HIV gene expression and the overexpression of NF90 had already been shown to enhance HIV gene expression in co-transfection experiments [12,18]. We initially sought to examine the effect of NF90 expression on HIV infection. The overexpression of NF90 increased HIV infection in both HIV-Luc infection assays (Figure 2A) as well as single cycle virus production assays (Figure 2C). Similar to NF45, NF90 has also been implicated in the replication of several RNA viruses [34,35], and we again hypothesized that NF90 may be eliciting its effects via RNA binding. Two deletion mutants predicted to disrupt the RNA binding function of NF90 were created (Figure 1): one contained an in-frame deletion of the two DRBMs (ΔDRBM) and the second was a large C-terminal deletion that deleted both DRBMs and the RGG motif (ΔCT). Although the expression of the mutants was not as robust as wild-type NF90, neither mutant significantly boosted HIV-Luc infection compared to control transfected cells (Figure 2A). These data suggested that similar to NF45 the RNA binding function of NF90 was required for the enhancement of infection.

Notably NF45 binds NF90 via its DZF motif and enhances NF90 activities [36,37]. Since both NF45 and NF90 enhanced HIV infection, we next tested if overexpression of both factors together would further enhance infection. To our surprise, robust expression of both proteins did not provide any substantial boost to infection levels compared to NF45 or NF90 alone (Figure 2A). These data suggested that the factors may be acting through the same mechanism or pathway that is saturated by overexpression of either protein.

**Figure 3:**
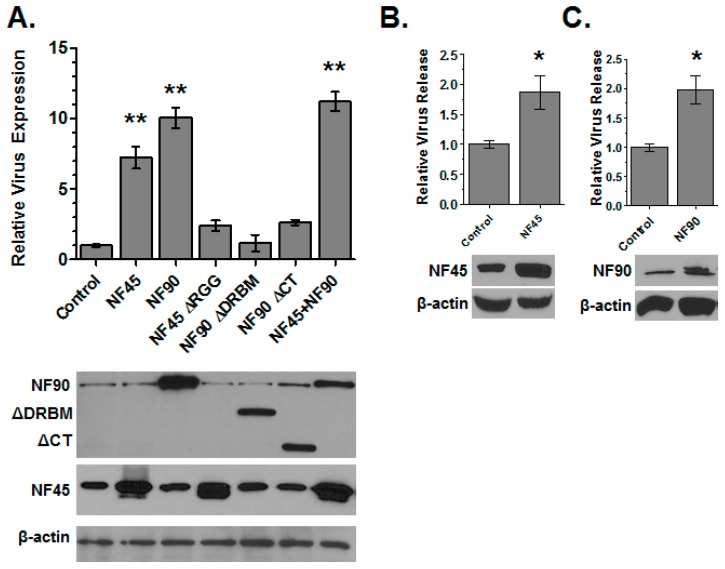
NF45 and NF90 potentiate HIV gene expression. (**A**) Expression assays. HIV-Luc plasmid was co-transfected with indicated expression plasmids or empty pcDNA3.1 as a control. At 48 h post transfection, cells were harvested and gene expression quantified by Luc assay, which was normalized to protein concentration. Protein overexpression was confirmed by immunoblot using β-actin as a control (**B**,**C**) Virus production assays. Cells were co-transfected with pNLX molecular clone and NF45 (**B**); NF90 (**C**); or pcDNA3.1. The next day the media was changed, and the cells incubated an additional 8 h before the supernatants were collected. Virus release was measured by *in vitro* reverse transcriptase assay and are presented relative to the control group. Protein expression was monitored by immunoblot using β-actin as a loading control (bottom panels). Plots show the total data from three independent experiments with triplicate transfections; blots are representative of all three experiments.

Any change in the level of infection could result from either a boost in the level of the early steps of virus replication (e.g., entry, reverse transcription, nuclear import, or integration) or higher gene expression after integration. To test if NF45 was affecting HIV gene expression, we performed co-transfection experiments with the wild-type and ΔRGG NF45 expression vectors and pNLX-Luc plasmid. Co-expression of NF45 resulted in a near eight-fold increase in Luc expression, whereas the ΔRGG mutant only slightly increased HIV expression (Figure 3A). The increase in gene expression was confirmed by measuring virus production from cells co-transfected with NF45 and the full-length pNLX HIV-1 molecular clone by measuring reverse transcriptase (RT) activity released into cell supernatants. Co-transfection of NF45 with pNLX resulted in a two-fold increase in virus production compared to control cells (Figure 3B). Combined, these data indicated that NF45 increases HIV infection by promoting gene expression and the effect requires the RNA binding domain of NF45.

Next we performed the co-transfection assays with NF90, ΔDRBM and ΔCT. Similar to the previous observations [18], NF90 significantly increased viral gene expression compared to control transfected cells as measured by Luc production (Figure 3A) or virus produced from cells (Figure 3C). However, deletion of the RNA binding domains of NF90, in either the ΔDRBM or ΔCT mutant, ablated the ability of NF90 to enhance virus production (Figure 3A). Once again, co-transfection of NF45 and NF90 together did not enhance virus expression substantially compared to either factor alone. Combined these data demonstrate that the ability of both NF45 and NF90 to enhance HIV virus production requires their RNA binding domains.

**Figure 4:**
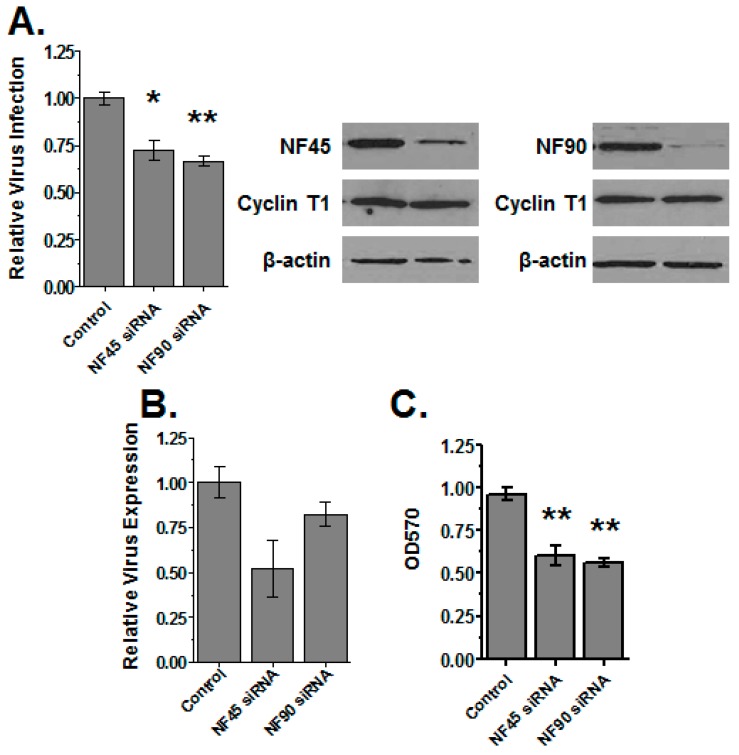
Knockdown studies of NF45 and NF90. (**A**) Cells were pre-transfected with small interfering RNAs (siRNAs) against NF45, NF90 or with scrambled siRNAs as a control. After 24 h cells were infected with VSVg-pseudotyped HIV-Luc reporter virus. At 24 hpi, cells were harvested and infection quantified by Luc assay and normalized to protein concentration. Knockdown was confirmed by immunoblot using β-actin as a control (panels at right); (**B**) The effect of knockdown on HIV gene expression. HIV-Luc plasmid was co-transfected with indicated siRNAs. After 24 h the cells were harvested and gene expression quantified by the amount of Luc produced relative to total protein concentration; (**C**) MTT assays. Cells transfected with siNF45, siNF90 and scramble siRNA as control were tested for viability by MTT assay after 24 h. Plots show the total data from three independent experiments with triplicate transfections; blots are representative of all three experiments.

It was reported that the knockdown of NF90 by RNA interference reduces cyclin T1 expression and decreases HIV gene expression [18]. However, knockdown of NF45 and NF90 has been shown to retard the cell growth [10], which would reduce viral expression. To revisit this issue, we knocked down NF45 or NF90 in 293T cells using siRNAs and performed virus infection and production assays as well as MTT assays to monitor cell viability. As shown in Figure 4A, in our hands virus infection was modestly, but not substantially reduced by knockdown of either NF45 or NF90. Virus gene expression was similarly impacted in co-transfection assays (Figure 4B). However, MTT assays revealed that knockdown of either NF45 or NF90 reduced cell health (Figure 4C), suggesting a mechanism for the loss of infection or production. Immunoblots confirmed the expected reductions in NF45 and NF90 expression. We also examined the levels of cyclin T1 in the knockdown cells (Figure 4A, blots). Neither the depletion of NF45, nor, in contrast to previous reports, NF90 substantially changed the levels of cyclin T1 expression in our experiments.

**Figure 5:**
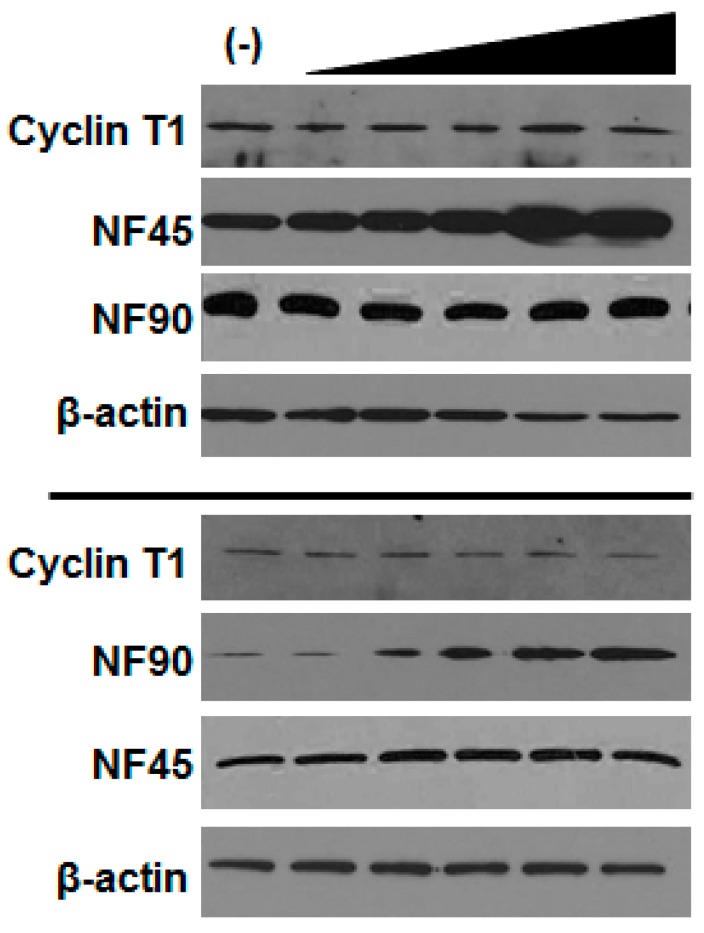
Cyclin T1 expression in cells over-expressing NF45 and NF90. 293T cells were transfected with increasing amounts of NF45 (top panels) and NF90 (middle panels). After 24 h, cells were harvested, normalized for protein concentration, and immunoblotted for indicated factors. Data are representative of duplicate experiments.

To further assess if cyclin T1 was contributing to the positive effect of NF45 or NF90 on HIV gene expression, we tested if cyclin T1 levels were altered in NF45 or NF90 over-expressing cells. To do this, we transfected increasing amounts of either NF45 or NF90 and examined the expression of cyclin T1 by immunoblot. As shown in Figure 5, we did not observe a change in the expression of cyclin T1 upon overexpression NF45 (top panels) or NF90 (bottom panels). Therefore, we concluded that the enhancement of HIV infection was not due to an increase in the expression of cyclin T1 resulting in increased promoter activity. We also probed the expression of NF90 upon NF45 overexpression and NF45 upon NF90 overexpression. Similar to our initial experiments (Figure 2A and Figure 3A), the overexpression of either factor did not appear to affect the expression of the other.

### 3.2. NF45 and NF90 Interact with HIV RNA

**Figure 6:**
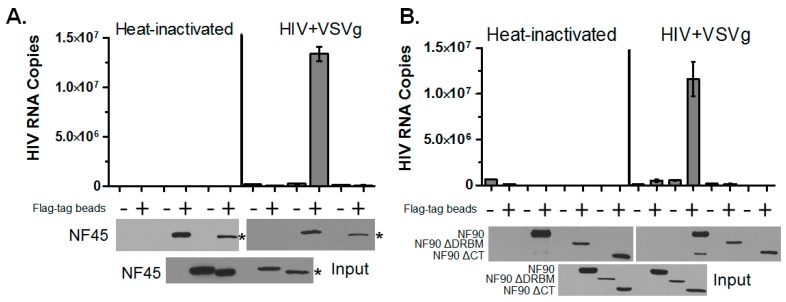
NF45 and NF90 interact with HIV RNA. (**A**,**B**). Cells were infected for 24 h with Heat inactivated virus or HIV+VSVg as indicated and protein-RNA complexes isolated with indicated antibodies ((−) = isotype control beads and (+) = flag tag beads). Immunoprecipitates were digested with PK prior to RNA isolation and HIV binding detected by real-time PCR using late RT primers. Immunoblots were used to confirm the expression (input) and capture (output) of proteins (* denotes NF45 ΔRGG).

Both NF45 and NF90 are promiscuous RNA binding proteins. NF45 binds single stranded RNA, whereas NF90 contains two double-stranded RNA binding motifs [37,38]. NF90 has been shown to have some binding selectivity as it has been shown to selectively repress the translation of RNAs bearing AU-rich elements [39]. Since the RNA binding domains of both proteins were required for the enhancement of HIV expression, we hypothesized that both proteins were interacting with the HIV RNA. To test this we performed IP-RT-PCR assays in infected cells. For these experiments, cells were pre-transfected with FLAG-tagged constructs and infected with HIV+VSVg for 24 h. The cells were lysed and immunoprecipitated with anti-FLAG beads. The immunoprecipitates were proteinase treated, and RNA isolated by acid phenol: chloroform extraction. The capture of HIV RNA was detected by real-time RT-PCR using *gag*-specific primers (Figure 6). Numerous controls were used to confirm the specificity of binding, including non-transfected cells, cells inoculated with heat-inactivated virus, and immunoprecipitation with an isotype control antibody. The results showed that both NF45 (Figure 6A) and NF90 (Figure 6B) interacted with HIV RNA. As expected, deletion of the RNA binding domains of either protein resulted in a loss of interaction with HIV RNA.

**Figure 7:**
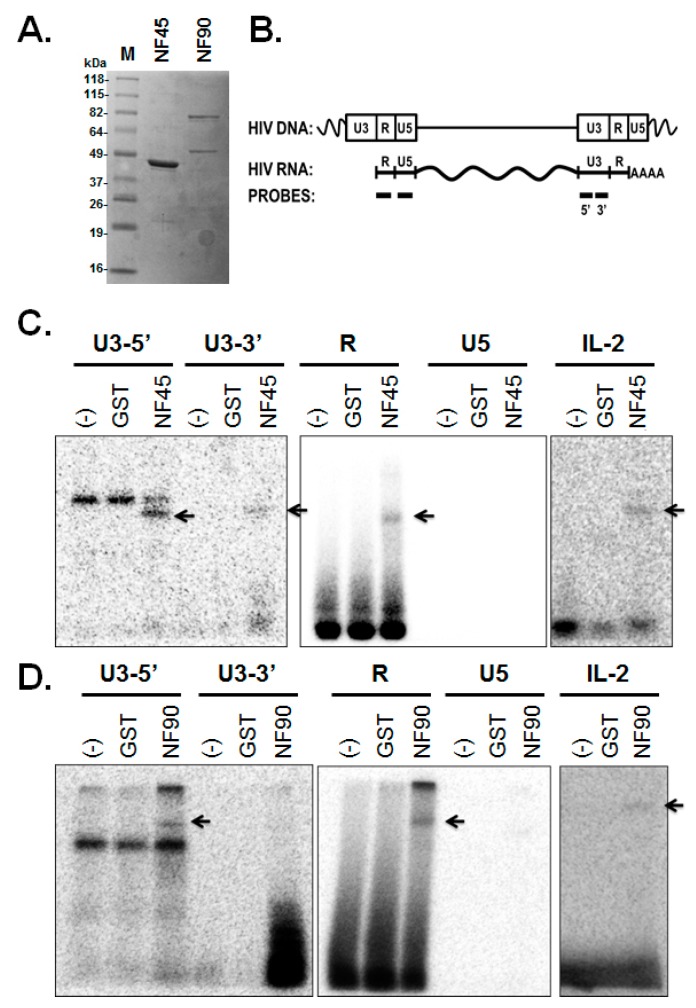
*In vitro* RNA cross-linking assays. (**A**) Expression of purified rNF45 and rNF90 visualized by SDS-PAGE and staining with Coomassie blue; (**B**) Cartoon depiction of integrated HIV DNA and mRNA. Approximate locations of RNA probes used in cross-linking assays are shown at bottom (**C**) *In vitro* RNA binding assays with rNF45 (**C**) and rNF90 (**D**). Recombinant proteins were incubated with radiolabeled RNA probes to regions indicated at top (IL2 3′ UTR was used as a positive control). Samples were cross-linked by UV exposure and treated with RNase A prior to separation by SDS-PAGE. Gels were imaged using a phosphorimager. Arrows indicate expected migration for HIS-tagged NF45 (**C**) and NF90 (**D**) proteins.

NF45 and NF90 have been shown to interact with other RNA binding proteins through binding to RNA. For example, the interaction of NF90/110 with the RNA-editing factor, adenosine deaminase acting on RNA (ADAR) is bridged by dsRNAs [40,41]. Another recent proteomic analysis of NF90 and NF110 binding proteins in mouse brain found that NF90 and NF110 interact with numerous hnRNPs, including A/B, A2/B1, A3, D, Q, as well as polypyrimidine tract-binding protein-associated-splicing factor [42]. Therefore it is possible that the NF45 and NF90/110 might interact with HIV RNA indirectly by binding other RNA binding proteins. To determine if NF45 or NF90 bound directly to HIV RNA, we performed *in vitro* cross-linking assays. Recombinant, HIS-tagged NF45 and NF90 were expressed in *E. coli* and purified by affinity chromatography (Figure 7A). Since both NF45 and NF90 may enhance the transcription of the HIV promoter activity, it suggested that the proteins may bind the 5′ end of the HIV RNA. Therefore, the RNA probes were designed to cover the LTR region of HIV-1, including the R and U5 regions (Figure 7B). We also constructed two probes covering the U3 region of the LTR which is present only in the 3′ end of the HIV mRNA. RNA probes were made by *in vitro* transcription and labeled with [^32^P]-UTP. After incubation at 37 °C, protein-radiolabeled RNA complexes were UV-crosslinked, digested by RNase, separated by SDS-PAGE, and imaged using a phosphorimager. In all experiments the 3′ UTR of the IL-2 mRNA was used as a positive RNA probe control [43], and purified GST protein was used as a negative input protein control. As seen in Figure 7C, we detected the interaction of NF45 with RNAs from the U3 and TAR regions of the HIV LTR (arrows), but not the U5 region. The non-specific bands may represent incompletely digested RNAs or cross-linking to background proteins in the protein preparations. NF90 cross-linked to RNAs from the 5′ half of U3 and the TAR region (Figure 7D), but not the 3′ half of U3 or the U5 regions. These results indicate that NF45 and NF90 bind HIV RNA, but the binding of multiple regions of the RNA suggested that the interactions were not sequence specific.

### 3.3. NF90 Stabilizes HIV LTR-Containing RNA in Vivo

**Figure 8:**
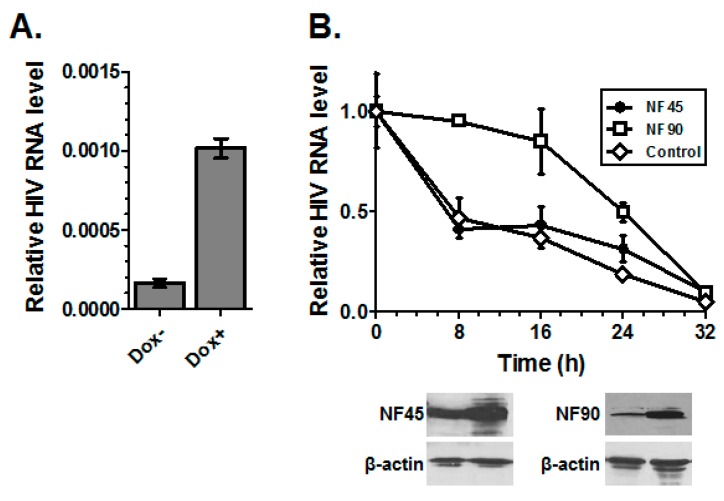
NF90 stabilizes the HIV RNA. (**A**) Inducible expression of HIV RNA. Transcription of HIV RNA in doxycycline inducible cell line (**B**) RNA stability assay. HeLa-HIV RNA cells were transfected with empty vector, pCMV-NF45 or pEF-NF90. After 24 h doxycycline was added to each well to induce the expression of HIV RNA. At 12 h post induction the cells were washed three times with warm cDMEM and returned to normal media to halt transcription. RNA was harvested at the indicated times and quantified by real-time RT-PCR. Results were normalized to the level of 18S RNA. Error bars represent standard deviation of triplicate PCR reactions.

Protein expression can be affected by many factors including the half-life of the mRNA. NF90 is known to stabilize the interleukin-2 mRNA by binding to the AU-rich element located within IL-2 mRNA 3′-UTR [43]. The phosphorylation of NF90 Ser647 is critical for this effect [44]. Since we observed that NF45 and NF90 bind HIV RNA, we sought to determine if either factor affected the stability of the RNA. Treatment with the RNA transcription inhibitor actinomycin D (ActD) is a widely used approach to study RNA decay, but low levels have been shown to upregulate HIV expression due to hyper-phosphorylation of C-terminal domain of RNA polymerase II [45,46]. Therefore, to measure HIV RNA decay we constructed a minimal inducible construct containing the HIV TAR and R3 RNA regions under control of tetracycline response element (pTRE-LTR). The construct was stably transfected into HeLa Tet-On^®^ 3G Cell Line and the expression of the HIV RNA element was induced by addition of doxycycline (Dox; Figure 8A). Thus, upon removal of Dox RNA transcription ceases and the decay rate of the RNA could be measured by real-time RT-PCR. To test if NF45 or NF90 impacted RNA stability, we transfected NF45 and NF90 24 h prior to the addition of Dox. After 12 h of Dox treatment the cells were washed and RNA then harvested at 0, 8, 16, 24 and 32 h post washout. The level of HIV RNA was measured by real-time PCR and normalized to the level of 18S ribosomal RNA (Figure 8B). The half-life of LTR RNA in mock-transfected cells was approximately 8 h. The overexpression of NF45 did not alter the decay rate of the RNA, but the overexpression of NF90 substantially increased the half-life of the RNA to approximately 24 h. These results suggest that one mechanism by which NF90 increases virus production may be stabilization of the HIV RNA.

## 4. Discussion

HIV utilizes host factors to facilitate replication and it would be expected that an increased expression of certain factors might potentiate virus infection. Identifying these factors may point toward pathways critical for virus replication that can be exploited for inhibitor development. We identified NF45 and NF90 in a previous proteomic screen [5]. Previous studies demonstrated that the knockdown of NF90 impaired HIV gene expression [18]; however, it has been shown that the knockdown of NF90 significantly impacts cell growth [10]. Moreover, NF90 knockout mice die within 12 h due to neuromuscular respiratory failure [11]. Therefore, it is possible that loss of cell viability caused the reduction in HIV infection irrespective of protein function. Indeed, in our experiments, the loss of infection upon knockdown of either NF45 or NF90 correlated with a loss of cell viability. Therefore the major focus of these studies was to examine the effects of positive expression of these proteins. We found that NF45 and NF90 potentiated HIV infection by promoting HIV gene expression. Notably, neither overexpression nor knockdown of NF45 or NF90 appeared to affect cyclin T1 expression in our studies, although further quantitative studies are necessary to definitively address this issue. The RNA binding domains of both proteins were required to potentiate HIV expression. Both NF45 and NF90 were found to bind HIV RNA *in vitro*, but the interactions appeared to be non-specific. Finally, overexpression of NF90, but not NF45, slowed the decay of an RNA containing HIV LTR elements. Combined these data indicate that both NF45 and NF90 are positive regulators of HIV transcription. NF90 may also upregulate HIV gene expression by stabilizing the HIV RNA. The role of NF45 RNA binding remains unclear. Interestingly, overexpression of both proteins did not have an additive effect on HIV gene expression. This suggests both proteins may act independently in the same pathway. Alternatively, the addition of one or the other factor may be sufficient to saturate effector sites on the HIV RNA or interactions with cellular factors that stimulate HIV gene expression [37]. Further studies with mutants known to disrupt the NF45-NF90 interface would clarify this issue [9,37].

The pleiotropic effects of NF45 and NF90 on HIV expression were not surprising as these are multifunction nuclear proteins that regulate cellular gene expression at different levels [47,48,49]. These functions include DNA damage repair mediated by DNA-PK [7], RNA translation [39,50] and microRNA biogenesis [51,52]. Indeed, NF45 and NF90 have been shown to alter the replication cycles of a number of viruses. NF90 interacts with the 5′ end of the hepatitis C virus RNA and is necessary for efficient virus replication [53]. NF90 binds the 3′ end of dengue virus RNA and potentiates virus replication [35]. The overexpression of NF90 inhibits influenza virus replication during the early phase of infection through an interaction with the viral nucleoprotein [34]. NF45 accumulates at sites of replication of infectious bursa disease virus (IBDV) and it may negatively regulate IBDV replication as siRNA knockdown of NF45 enhances replication [33]. It has also been found that knockdown of NF45 or NF90 restores the expression of the p53 and p21 proteins in cervical carcinoma cells infected with high-risk human papillomaviruses (HPVs) [47]. It has also been shown that NF90 is a component of stress granules, which possess antiviral activity [54].

While our data suggest that NF45 and NF90 increase HIV production through interactions with HIV RNA, we cannot exclude the possibility that they may control the expression of other host factors and lead to an enhancement of infection. Proteomic analyses of the NF90/110 interactome show interactions with a number of RNA processing proteins, including numerous Heterogeneous nuclear ribonucleoproteins (hnRNPs) [42]. NF90 has also been shown to interact with the anti-viral factor PKR, which is known to be inhibited during HIV infection [55,56]. It could be possible that the overexpression of NF90 alters PKR activity in a manner that enhances HIV infection. The transcription factor YY1 is another HIV factor that interacts with NF90 [57,58]. YY1 has been shown to repress LTR activity; therefore, NF90 overexpression may sequester this factor and lead to increased viral gene expression. Further studies that compare the interactomes of NF45 and NF90 in uninfected and HIV infected cells are needed to address this issue and identify other factors that may alter HIV gene expression.

The NF90ctv C-terminal variant of NF90 was shown to reduce HIV gene expression by competing with Tat for binding to TAR RNA [19]. Our data confirmed that NF90 binds TAR, but show clearly that overexpression of NF90 potentiates HIV gene expression. Combined with previous data [18], we believe these results convincing show that NF90 is a positive regulator HIV infection. It may be that the alteration in the C-terminus of NF90ctv may create a dominant negative mutant of HIV transcription.

Lastly, in contrast to Hoque *et al.* we found that NF45 potentiates HIV transcription and infection using two independent experimental systems. This discrepancy may be due to the variability in methodology including the cell lines (HeLa *vs.* 293T), strains of virus used, or the nature of the NF45 expression vectors used. NF45 has been shown to interact with the RNA of several viruses. In addition to IBDV, NF45 was found to bind the IRES of rhinovirus type 2 and inhibit translation [59]. It has also been shown to interact with the hepatitis C capsid protein via a RNA intermediate [60], although the functional consequence of this interaction is unknown. We show that NF45 enhances HIV infection by upregulating HIV gene expression, and we observed that it bound HIV RNA but did not alter its stability. We do not yet know how NF45 increases HIV infection. It may potentiate translation, or activate gene expression through a cellular pathway. Further studies will be needed to delineate the role of NF45 in HIV replication.
